# Premature Burial of Medical Papers

**Published:** 1875-04

**Authors:** 


Premature Burial of Medical Papers.—Many of these papers possess a well-developed vitality, and die mainly because they are not placed in an atmosphere congenial to their growth, or they are prematurely interred in unknown graves, from absolute lack of appreciation of their inherent mental or physical force. Of this class are the various literary productions which, under the name of "Transactions" of National and especially of State organizations, find a temporary resting-place, in undisturbed repose, on the practitioner's book-shelf, to become, in time, a matter of interest to the ragman. The contributions they contain are literally buried alive, and soon pass into undeserved oblivion, the author alone dropping the regretful tear. There is hardly a volume of "Transactions," in these days of advancement, but contains some paper worth preserving in the columns of a medical journal, but, in the present system of publishing, the profession at large is but little benefited. We seldom see extracts in medical serials from these annual *omnium gathera*, and when they do appear they have a decidedly *post mortem* odor, on account of the still-born character of the original paper. As we have already intimated in other phraseology, the assistance of the accoucher and the nurse is alone needed to insure the prolonged healthy existence of these literary bantlings; the resurrectionist has but slender hopes of success. The whole system is radically wrong, and should be speedily amended. Far better would it be to inaugurate at the earliest available moment a new rule in this respect, and in the pages of a medical journal of respectable circulation—if need be by authoritative selection on the part of the State society itself—give deserving contributions a prominence which they have never yet possessed.—*Medical Record.*

